# Sensitive RP-HPLC method with fluorimetric detection for concurrent quantification of emtricitabine, Daclatasvir and Ledipasvir in human urine

**DOI:** 10.1038/s41598-025-07401-y

**Published:** 2025-07-01

**Authors:** Aya A. Youssef, N. Magdy, Lobna A. Hussein, A. M. El-Kosasy

**Affiliations:** Pharmaceutical Analytical Chemistry Department, Faculty of Pharmacy, Ain Shams University, Organization of African Unity Street, Abassia, Cairo, 11566 Egypt

**Keywords:** Emtricitabine, Daclatasvir, Ledipasvir, Bioanalytical validation, HIV/HCV co-infection, Analytical chemistry, Medicinal chemistry, Infectious diseases, Public health

## Abstract

Co-infection with hepatitis C virus (HCV) in human immunodeficiency virus (HIV) patients is common and has a poor prognosis leading to many complications. The anti-HIV drug emtricitabine (FTC) is co-administered with two direct acting anti-HCV drugs; daclatasvir (DAC) and ledipasvir (LDV). The three drugs are simultaneously determined in human urine for the first time by a validated, simple and sensitive RP-HPLC with programmed fluorescence detection. The column used is Exsil 100 ODS C18 column (250 × 4.6 mm, 5 μm). The used mobile phase is acetonitrile: methanol: 0.01 M ammonium acetate buffer, pH 4.5 in ratio (20: 60: 20) in isocratic mode pumped at flow rate 1 mL/min. The proposed method is successfully validated according to FDA bioanalytical validation guidelines. The calibration curves are linear over the ranges (500-15000, 1–50 and 10–100 ng/mL) with average recoveries (97.9-99.54%, 98.78-104.17% and 98.49–100.96%) for FTC, DAC and LDV, respectively. The intraday and inter-day accuracy and precision results are within the acceptable limits. Stability assays reveal that the three studied drugs were stable during preparation, injection and storage. The method can be applied for the quantification of the three drugs co-administered to HIV/HCV co-infected patients’ urine which aids in therapeutic drug monitoring and dosage adjustment for chronic patients.

## Introduction

Acquired immunodeficiency syndrome (AIDS) is still regarded as a challenging global public health issue. It is caused by human immunodeficiency virus (HIV) infection. HIV infections often develop multiple complications and comorbidities. At the end of year 2021, the Joint United Nations Program on HIV/AIDS (UNAIDS) reported that the prevalence of HIV among adults was 0.7% with 650,000 AIDS-related deaths around the world^1^. Infection with hepatitis C virus (HCV) is the most common co-infection in HIV patients since both infections share similar routes of transmission^2^. Additionally chronic HCV infection is a major public health problem affecting 1% of the world population^3^. HCV causes chronic liver disease and cirrhosis which if untreated results in hepatocellular carcinoma leading to significant mortality. Thus, patients co-infected with HCV and HIV are susceptible to significant complications and rapid disease progression compared to HCV mono-infected patients. As a result, treatment of HCV/HIV co-infection is complicated and requires early intervention with long-term combined treatment protocols^4,5^.

HIV/HCV combined treatment protocols include antiretroviral therapy (ART) for HIV treatment and direct-acting antivirals (DAAs) against HCV^6^. In the last decade DAAs class replaced the old standard HCV treatment and proved better antiviral efficacy. Recently, the common HCV treatment protocols include one or more drugs from the DAAs such as sofosbuvir, ledipasvir and daclatasvir^7,8^. While the most common ART used against HIV are emtricitabine and tenofovir^2,4^. According to the patient’s condition certain combination drug regimens are applied for instance, emtricitabine used for long term therapy for HIV can be co-administered with DAC and LDV^6,9,10^. Therefore, HIV/HCV co-infected patients follow prolonged treatment protocols which necessitates close monitoring and follow-up to detect the drugs levels in biological fluids.

**Fig. 1:**
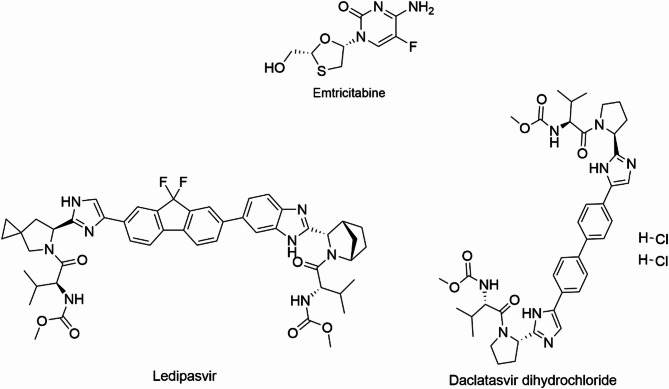
Chemical structures of emtricitabine, daclatasvir and ledipasvir.

Emtricitabine (FTC) is used in the treatment of HIV in combination with other antiretroviral therapy (ART), its chemical structure is depicted in Fig. 1. FTC is nucleoside reverse transcriptase inhibitor (NRTI), it inhibits reverse transcriptase–catalyzed mRNA-dependent DNA and thus ends the amino acid chain of generated viral DNA^11^. FTC is also a key component of pre-exposure prophylaxis (PrEP) for HIV prevention used for HIV-negative persons at risk of infection^12^. A number of published HPLC methods have been applied for the analysis of FTC in combination with other drugs in dosage form^13–16^. All the mentioned HPLC methods used UV or PDA detection. But only one method has been reported for FTC determination by RP-HPLC using fluorescence detector in plasma^17^. Noticeably, the literature is devoid of any HPLC method measuring FTC in human urine although pharmacokinetic data report that FTC is detectable in urine unchanged^18^.

Both daclatasvir (DAC) and ledipasvir (LDV) belong to the direct-acting antivirals (DAAs) class used for HCV infection treatment (Fig. 1)^8,19^. DAC and LDV are selective inhibitors of HCV non-structural 5 A (NS5A) protein. NS5A protein is vital for HCV replication and cellular signaling pathways regulation. They are commonly used in HCV treatment regimens and co- administered with other drugs such as sofosbuvir and/or ribavirin^7,8,20^.Few HPLC-DAD methods have been published for determination of DAC or LDV in dosage forms^21–24^. Whereas, DAC and LDV were determined simultaneously with other DAAs or ribavirin either in bulk or in dosage forms by a number of HPLC methods with UV detection^25–29^. A review of the published analytical methods indicates that no HPLC methods employing fluorescence detection have been reported for the simultaneous determination of FTC, DAC and LDV in urine. Only a single method has been documented for the determination of DAC alone, and that was limited to pharmaceutical dosage form^30^.

In addition, the three studied analytes exhibit native fluorescence, rendering them suitable for fluorescence detection^17,31^. Fluorescence detection offers some advantages over UV detection: it provides high sensitivity, allowing detection limits in the nano-range, and high selectivity, with minimal interference from non-fluorescent components. Hence, fluorescence detection is well-suited for the determination of drugs in biological matrices, owing to its high sensitivity and selectivity at specific excitation and emission wavelengths, which significantly minimizes matrix effects. Accordingly, analyte quantification using a fluorescence detector is considered beneficial due to its sensitivity and selectivity.

It was reported that FTC is primarily eliminated by kidneys and excreted predominantly in urine; 86% of the administered dose is excreted in urine^18,32,33^. While DAC and LDV are excreted unchanged in patients’ urine as percentages 6.6% and 1% respectively^19,34^. Biological analysis of drugs in urine specimens is simpler relative to other biological fluids. Urine collection is non-invasive, particularly suitable for all age groups and requires minimal preparations prior to analysis^33,35,36^. Despite the presence of FTC, DAC and LDV in detectable concentrations in patients’ urine and the ease of using urine specimens in drugs analysis, till now there is no published HPLC method in literature for determination of the three analyzed drugs simultaneously in human urine. Hence, this study shows the first, sensitive and simple HPLC method with fluorescence detection for the determination of FTC, DAC and LDV in human urine reaching ultrasensitive concentrations.

It is worth mentioning that, applying an HPLC method is simpler, cost effective with lower maintenance and operational expenses in contrast to LC-MS/MS that is not accessible in some labs especially in developing countries. Also, HPLC is well-established in clinical settings for routine therapeutic drug monitoring (TDM) and provides reliable sensitivity and selectivity for many analytes. Considering the clinical role of TDM for optimizing antiviral therapy, particularly in special populations such as co-infected patients with HIV/HCV aiming to better clinical outcomes. The developed sensitive and selective HPLC method for the simultaneous determination of FTC, DAC, and LDV in urine using fluorescence detection is highly warranted for treatment optimization.

## Experimental

### Chemicals and reagents

Emtricitabine (99.6% purity) was purchased from Sigma-Aldrich (USA). Daclatasvir (99.8% purity) and ledipasvir (99.6% purity) were obtained from ALSACHIM (France). All utilized solvents were HPLC gradient grade (99.9% purity). Water, methanol and acetonitrile were bought from Sigma-Aldrich (USA). Ammonium acetate and chloroform were purchased from FischerScientific (Cornell Lab, Cairo, Egypt). Urine samples were collected from healthy male adult (age 30–44) human volunteers.

### Instrumentation

The chromatographic separation and analysis of the studied analytes was conducted using VWR^®^ Hitachi Chromaster HPLC-DAD-FL system equipped with autoinjector, quaternary pump, vacuum degasser, Hitachi Chromaster 5430 diode array detector and Hitachi Chromaster 5440 fluorescence detector (Japan). The used stationary phase was reversed phase Dr. Maisch GmbH (Ammerbuch, Germany) Exsil 100 ODS C18 column (250 × 4.6 mm, 5 μm). Jenway digital pH meter was used to adjust and determine pH of buffer solution. VELP Scientifica RX3 vortex mixer, BOECO centrifuge SC-8 and Eppendorf^®^ concentrator 5301 were used for sample preparation.

### Chromatographic conditions

The used mobile phase was acetonitrile: methanol: 0.01 M ammonium acetate buffer, pH 4.5 in ratio (20: 60: 20) pumped at flow rate 1 mL/min. The column was set at room temperature (25 ºC) during the chromatographic separation. The programmed fluorescence detector (FLD) was set at different excitation (λ_exc_) and emission (λ_em_) wavelengths specific to each determined drug throughout the 15 min of the run time as illustrated in Table 1. The injected volume by the autoinjector was 20 µL. Pre-washing of the column was done daily for 30–45 min prior to sample injection by water and methanol in different ratios, or as needed, to remove retained carryover if any and to extend column lifetime. Conditioning for 10 min with the mobile phase was done between runs before each analysis to ensure column equilibration and stable baseline.

**Table 1:** Programmed FLD setup showing λ_exc_, λ_em_ of the studied drugs.

Time interval (min)	Excitation wavelength (λ_exc_) (nm)	Emission wavelength (λ_em_) (nm)	Separated drug
0–4	270	350	Emtricitabine
4–7	300	380	Daclatasvir
7–15	340	420	Ledipasvir

### Preparation of calibration standards and quality control samples

Stock solutions (500 µg/mL) of FTC, DAC and LDV were prepared in methanol and stored at 4 ºC. On the day of analysis, working solutions of each analyte were prepared with the following concentrations 250, 10 and 100 µg/mL for FTC, DAC and LDV respectively. A mixture of the analytes with lower concentrations were prepared in nine 10 mL volumetric flasks (6 calibrators and 3 QC samples) by accurately transferring appropriate volumes from each working solution. The prepared concentrations of the mixture denoted as C_mix_ are stated in Table 2. From each volumetric flask 100 µL were accurately transferred to 900 µL urine for spiking to reach final volume 1000 µL spiked urine with final concentrations denoted C_f_. Urine samples for spiking were collected after the approval of the Research Ethics Committee at Faculty of Pharmacy, Ain Shams University, Cairo, Egypt, with approval number: REC#285. All the procedures were done in accordance to the committee’s guidelines and after obtaining informed consents from all addressed subjects.

**Table 2:** Preparation of calibrators and quality control samples for FTC, DAC and LDV.

Prepared samples	Volume of urine	Concentration of each analyte in the prepared mixture before spiking (C_mix_) (ng/mL)			Final volume after spiking	Final concentration of each analyte in urine (C_f_) (ng/mL)		
		FTC	DAC	LDV		FTC	DAC	LDV
Calibrators	900 µL	5,000	10	100	1000 µL	500	1	10
		20,000	100	200		2000	10	20
		50,000	200	400		5000	20	40
		90,000	300	600		9000	30	60
		120,000	400	800		12,000	40	80
		150,000	500	1000		15,000	50	100
QCL		15,000	30	300		1500	3	30
QCM		70,000	250	500		7000	25	50
QCH		130,000	450	900		13,000	45	90

C_mix_: Concentration of the drug in the mixture, C_f_: Final drug concentration in spiked urine, QCL: Quality Control sample Low, QCM: Quality Control sample Middle, QCH: Quality Control sample High.

### Urine sample extraction and preparation

FTC, DAC and LDV were extracted from spiked urine samples by liquid-liquid extraction. Three mL chloroform were added to 1 mL spiked urine and vortexed by RX3 VELP vortex mixer for 10 s. Then centrifugation at 2,800 RCF using BOECO centrifuge SC-8, for 15 min to separate the immiscible organic layer from the spiked urine aqueous layer. The denser organic chloroform layer was carefully transferred into clean Eppendorf^®^ microcentrifuge tube to be evaporated for 20 min till dryness using Eppendorf^®^ concentrator 5301 under vacuum at 25 °C. The residue was reconstituted in 1 mL of the mobile phase, vortexed for 10 s and filtered through 0.45 μm filter. After filtration, 20 µL were injected into the HPLC system.

### Method validation

The method was validated in accordance with the US Food and Drug Administration guidance for bioanalytical method validation^37,38^.

#### Selectivity

Six different blank samples of human urine from different volunteers (males, age 30–44) were prepared and analyzed. By comparing the chromatograms of the blank and that of the spiked analytes urine samples at LLOQ levels, any interference by the matrix can be assessed. The blank urine samples should be free of interference at the retention times of the analytes which means that, blank should be < 20% of the analyte response at the LLOQ.

#### Linearity

Spiked urine samples were prepared in the ranges of (500-15000 ng/mL, 1–50 ng/mL and 10–100 ng/mL) for FTC, DAC and LDV respectively as depicted in Table 2. They were injected under the mentioned HPLC conditions and areas of the resulting peaks analytes were calculated. The peak areas were plotted against their corresponding concentrations and the regression equations were calculated.

#### Accuracy and precision

Accuracy and precision of the assay were determined by analysis of the QC samples at four concentration levels (LLOQ, QCL, QCM and QCH). The intra-day (within-run) and inter-day (between-run) accuracy and precision were assessed by analysis of five replicates of QC samples within the same day and on three consecutive days. Precision was expressed as a percentage of relative standard deviation (%RSD).

#### Recovery

The recoveries of FTC, DAC and LDV from urine for three levels QC samples (QCL, QCM and QCH) were determined. They were calculated by comparing the peak areas of the human urine samples spiked by the analytes then extracted to that of the post extracted human urine samples spiked by the analytes at the same concentrations. Thus, the recoveries and the extraction efficiency of the method can be evaluated.

#### Stability

The stability of the analytes determined in human urine was evaluated by analyzing three replicates of QCL and QCH samples. By comparing peak areas of newly prepared spiked urine samples to that exposed to different conditions of storage and temperature. For freeze and thaw stability, samples were subjected to three freeze and thaw cycles for 72 h. Whereas, for benchtop stability the samples were left for approximately 8 h at ambient temperature. Ultimately, for processed sample stability the samples were kept in the autosampler for 24 h at ambient temperature to access if possible degradation of the sample might take place due to delay in injection.

#### Robustness

Robustness was evaluated by analyzing FTC, DAC and LDV after slight but intentional changes in the separation conditions. Flow rate was changed by (± 0.1 ml/min), buffer pH was changed by (± 0.2 units) also; organic strength in the mobile phase composition was varied by (± 2%) in methanol. The % relative standard deviation (%RSD) was calculated for the obtained recoveries of the analytes QCM before and after these changes.

## Results and discussion

This work aims to develop a new HPLC method to determine three co-administered drugs for the treatment of HIV/HCV co-infection. Determination of these drugs is challenging to evaluate patients’ conditions and drug adherence that ultimately impacts public health. To our information, this is the first reported HPLC method for simultaneous determination of FTC, DAC and LDV in human urine. Moreover, using fluorimetric detector renders the method sensitive to detect the mentioned drugs even in the nano-range concentrations. Additionally, all determinations were carried out in human urine which can be obtained easily by noninvasive procedures unlike other matrices. Consequently, the method is proposed to be applied in therapeutic drug monitoring for chronic HIV/HCV patients owing to its low cost, sensitivity and simplicity.

### Method development

#### Optimization of chromatographic conditions

A successful analysis of ternary mixture of FTC, DAC and LDV was developed using isocratic HPLC method with fluorimetric detection. The method conditions were optimized to achieve acceptable resolution, peak shape, sensitivity and short analysis time. Various trials were carried out to optimize the mobile phase composition aiming to achieve the best separation. Basically, water and acidic water were tried but weren’t suitable for separating FTC. Buffers such as phosphate and acetate were tested at different pH ranges (pH 4.0 to 8.5). Ammonium acetate buffer at pH 4.5 was selected based on the ionization states of studied analytes to achieve optimal resolution, symmetric peaks without tailing and appropriate retention times. The concentration of the buffer was optimized by trying concentrations range from 0.01 to 0.05 M and 0.01 M was the selected one. Generally, low-concentration buffers are preferred in HPLC to minimize detector interference, prevent salt precipitation and extend column lifetime.

Organic modifiers (methanol, acetonitrile or ethanol) were tried at various ratios with ammonium acetate buffer. Each of methanol and acetonitrile at 80% could separate the three analytes however, the resulted peaks in case of methanol were broad and in case of acetonitrile were eluted earlier and overlapped with early urine co-eluting peak but they are narrower compared to that of methanol alone. Thus, a mixture of both methanol, acetonitrile together with 0.01 M ammonium acetate buffer in ratio 60:20:20 was selected to achieve sharper, well-resolved peaks without interference.

The column temperature was kept at ambient temperature (approx. 25 ºC) throughout the separation. This is consistent with the majority of previously reported HPLC methods analyzing each of FTC, DAC and LDV all of which operated at ambient temperature, except one method for FTC determination increased the column temperature to 35 ºC^17^. This elevated temperature was tried in the present study however, it didn’t affect the separation significantly. Therefore, the column temperature was maintained at ambient temperature.

#### Sample extraction optimization

Urine samples are commonly used in drug testing due to their ease of collection and the relatively simple procedures required for analysis which is considered a noninvasive, low-cost testing alternative. In many cases, urine samples are diluted with water or a mixture of water and buffer, filtered then directly injected to HPLC column. This simplicity makes urine an attractive matrix for detecting drugs, as it minimizes the time and resources needed for sample preparation while still providing reliable results. However, some reports highlight that to achieve a more purified form of the drugs, urine samples may undergo extraction methods. Techniques such as protein precipitation or liquid-liquid extraction using specific organic solvents are employed to isolate the drugs from the urine matrix more effectively^39^. These methods help to enhance the accuracy and sensitivity of the drug detection process. Additionally, eliminating matrix peaks effect can prolong the lifetime of the used analytical column to reduce analysis cost in limited resources labs.

Both protein precipitation and liquid-liquid extraction as extraction procedures were tried. Better recoveries were achieved in case of using liquid-liquid extraction by chloroform solvent compared to other solvents such as, hexane, ethyl acetate and diethyl ether.

#### Fluorescence sensitivity

Fluorescence detection was used to detect the analytes in this mixture. Fluorimetric detection is characterized by being more specific to each analyte and sensitive compared to UV-detection, less expensive and more available with respect to mass spectrometry.

FTC exhibits excitation wavelength at 270 nm, emission wavelength at 350 nm and retention time 2.6 min. While DAC has retention time 4.8 min, excitation wavelength at 300 nm and emission wavelength at 380 nm. At retention time 9.9 min LDV was separated having excitation wavelength at 340 nm and emission wavelength at 420 nm.

Changing the excitation and emission wavelengths in a time-programming mode was employed to allow the quantitation of three analytes in the same chromatogram according to the retention time of each drug as shown in Table 1. The analytes were adequately separated under the previously indicated chromatographic conditions.

### Method validation

The proposed method was validated according to the FDA guidance for bioanalytical method validation^37,38^. The obtained parameters successfully met the defined acceptance criteria.

#### Selectivity

**Fig. 2:**
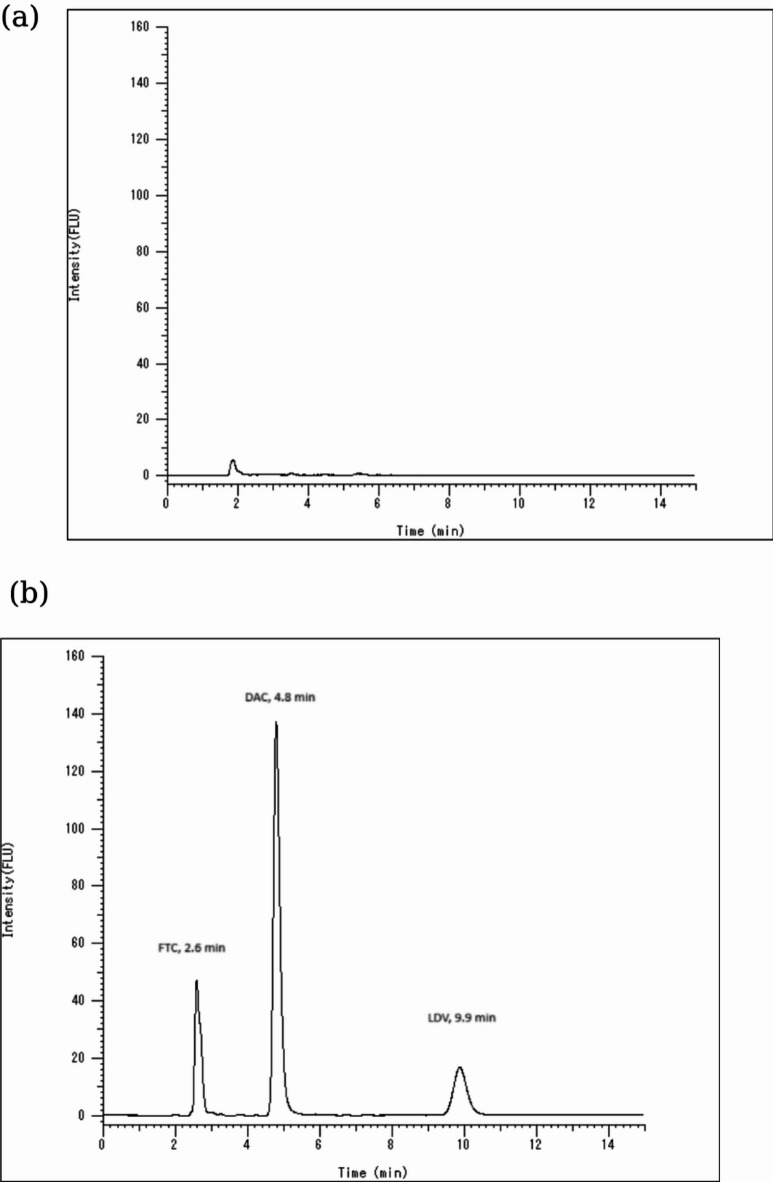
(**a**) HPLC chromatogram of blank human urine sample. (**b**) HPLC chromatogram of mixture containing FTC (5000 ng/mL) at λem 350 nm, DAC (45 ng/mL) at λem 380 nm and LDV (20 ng/mL) at λem 420 nm in extracted human urine samples.

The obtained chromatograms revealed good selectivity of the method. The blank human urine chromatogram in Fig. 2 (a) and the chromatogram of the spiked samples with the analytes shown in Fig. 2 (b) didn’t show any interfering peaks from the matrix at any of the retention times of the three analytes.

#### Linearity and sensitivity

The proposed method was linear for each analyte over the concentration ranges (500–15,000 ng/mL, 1–50 ng/mL and 10–100 ng/mL) for FTC, DAC and LDV respectively. The plotted calibration curves revealed correlation coefficients (r) more than 0.999 for each analyte as depicted in Table 3 together with other regression parameters.

These concentration ranges were selected according to the urinary excretion profiles of the three analytes FTC, DAC and LDV which are excreted in urine at 86%, 6.6%, and 1% of the administered dose, respectively^18,19,32–34^. Thus, the approximate expected urine concentrations for each drug are: for FTC 143 mg/L (dose 200 mg once daily), for DAC 3 mg/L (dose 60 mg once daily) and for LDV 0.75 mg/L (dose 90 mg once daily), assuming 1.2 L/day urine output as middle range for healthy adults^40^. However, it is impossible to inject these high concentrations into the HPLC because this might exceed the detector response limit resulting in inaccurate results, cause column overload and increase the risk of carryover. Therefore, the selected linear ranges of this proposed method are achieved by approximately 100-fold dilution of collected urine samples which is routinely done in clinical laboratories before urine samples injection into HPLC.

Sensitivity of the method was verified through getting acceptable accuracy and precision (within 20% deviation) for analytes’ LLOQ concentrations as mentioned in accuracy results. The LLOQ samples were 500, 1 and 10 ng/mL for FTC, DAC and LDV, respectively.

**Table 3:** Parameters obtained from emtricitabine (FTC), daclatasvir (DAC) and ledipasvir (LDV) linearity curves.

Parameter	Emtricitabine (FTC)	Daclatasvir (DAC)	Ledipasvir (LDV)
Linearity range (ng/mL)	500–15000	1–50	10–100
n	6	6	6
Slope	103	6536.9	1732.5
Intercept	− 1048.9	87,991	− 1659.6
Correlation coefficient (r)	0.9993	0.9995	0.9992
Lower limit of quantification (LLOQ) (ng/mL)	500	1	10

#### Accuracy and precision

The calculated %recoveries of each analyte by the developed method were demonstrated in Table 4, the intra-day accuracy values of the assay were between 99.13 and 102.12% with a precision of 0.65–2.95% for emtricitabine. For daclatasvir, the intra-day accuracy ranged from 99.63 to 102.45% with a precision of 0.61–4.57%. Regarding ledipasvir, the intra-day accuracy varied from 99.86 to 103.49% with a precision of 0.59–1.33%.

Whereas, results of inter-day accuracy and precision were illustrated in Table 4; emtricitabine inter-day accuracy values were 99.14-103.22% with a precision of 1.61–2.86%. While daclatasvir inter-day accuracy values were in the range 100.08-106.72% with a precision of 0.81–5.69%. For ledipasvir, inter-day accuracy values were 99.36-104.68% with a precision of 0.53–1.82%. All the results met the acceptance criteria stated by the FDA; results fell within ± 15% of nominal concentration except for the LLOQ where deviation within ± 20% was allowed^37,38^.

**Table 4:** Accuracy and precision for the HPLC determination of emtricitabine (FTC), Daclatasvir (DAC) and Ledipasvir (LDV) in human urine.

Intraday accuracy and precision						
Sample	Emtricitabine (FTC)		Daclatasvir (DAC)		Ledipasvir (LDV)	
	Conc. (ng/mL)	Mean %recovery* ± %RSD	Conc. (ng/mL)	Mean %recovery* ± %RSD	Conc. (ng/mL)	Mean %recovery* ± %RSD
LLOQ	500	102.12 ± 1.14	1	102.46 ± 4.57	10	103.49 ± 1.19
QCL	1500	99.78 ± 2.32	3	101.53 ± 3.95	30	101.39 ± 1.33
QCM	7000	99.13 ± 0.65	25	101.42 ± 3.04	50	99.86 ± 0.59
QCH	13,000	101.69 ± 2.95	45	99.63 ± 0.61	90	97.92 ± 0.97

Inter-day accuracy and precision						
Sample	Emtricitabine (FTC)		Daclatasvir (DAC)		Ledipasvir (LDV)	
	Conc. (ng/mL)	Mean %recovery* ± %RSD	Conc. (ng/mL)	Mean %recovery* ± %RSD	Conc. (ng/mL)	Mean %recovery* ±% RSD
LLOQ	500	103.22 ± 1.61	1	106.72 ± 1.35	10	104.68 ± 1.82
QCL	1500	102.42 ± 2.86	3	105.64 ± 5.69	30	101.48 ± 0.53
QCM	7000	99.30 ± 1.70	25	100.08 ± 1.33	50	99.36 ± 1.49
QCH	13,000	99.14 ± 1.99	45	100.51 ± 0.81	90	97.65 ± 1.54

*Mean of five determinations.

#### Recovery

The extraction efficiency of the method for the studied analytes was assessed by determining the recoveries of QC samples (QCL, QCM and QCH) as shown in Table 5. The extraction recovery values were 97.9-99.54% with %RSD 1.14–3.21% for emtricitabine. In case of daclatasvir extraction recovery values were 98.78-104.17% with %RSD 0.96–2.45%. For ledipasvir, extraction recovery values ranged from 98.49 to 100.96% with %RSD of 0.84–1.81%. These results indicate good extraction recovery by chloroform proving the efficiency of the used extraction method.

**Table 5:** Recovery results for HPLC determination of emtricitabine (FTC), Daclatasvir (DAC) and Ledipasvir (LDV).

Drug	Emtricitabine (FTC)		Daclatasvir (DAC)		Ledipasvir (LDV)	
Sample	Conc. (ng/mL)	Mean %recovery* ± %RSD	Conc. (ng/mL)	Mean %recovery* ± %RSD	Conc. (ng/mL)	Mean %recovery* ± %RSD
QCL	1500	98.68 ± 3.21	3	104.17 ± 2.45	30	100.96 ± 0.84
QCM	7000	97.90 ± 1.14	25	101.16 ± 2.01	50	99.86 ± 0.89
QCH	13,000	99.54 ± 3.47	45	98.78 ± 0.96	90	98.49 ± 1.81

*Mean of five determinations.

#### Stability

Freeze-thaw, bench top and processed sample stability results are illustrated in Table 6. Data revealed that %recoveries were in the accepted range ± 15% indicating that the QC samples were stable at the tested conditions.

**Table 6:** Stability results of emtricitabine (FTC), Daclatasvir (DAC) and Ledipasvir (LDV) in human urine under different stability assessment conditions.

Freeze-thaw stability**						
Drug	Emtricitabine (FTC)		Daclatasvir (DAC)		Ledipasvir (LDV)	
Sample	Conc. (ng/mL)	Mean %recovery* ± %RSD	Conc. (ng/mL)	Mean %recovery* ± %RSD	Conc. (ng/mL)	Mean %recovery* ± %RSD
QCL	1500	103.94 ± 1.01	3	104.83 ± 4.38	30	101.89 ± 0.87
QCH	13,000	98.21 ± 2.09	45	100.07 ± 1.13	90	98.81 ± 2.20

Bench top stability						
Drug	Emtricitabine (FTC)		Daclatasvir (DAC)		Ledipasvir (LDV)	
Sample	Conc. (ng/mL)	Mean %recovery* ± %RSD	Conc. (ng/mL)	Mean %recovery* ± %RSD	Conc. (ng/mL)	Mean %recovery* ± %RSD
QCL	1500	103.13 ± 0.77	3	103.96 ± 2.69	30	101.85 ± 0.62
QCH	13,000	98.54 ± 1.96	45	98.92 ± 1.44	90	97.82 ± 1.80

Processed sample stability						
Drug	Emtricitabine (FTC)		Daclatasvir (DAC)		Ledipasvir (LDV)	
Sample	Conc. (ng/mL)	Mean %recovery* ± %RSD	Conc. (ng/mL)	Mean %recovery* ± %RSD	Conc. (ng/mL)	Mean %recovery* ± %RSD
QCL	1500	103.23 ± 1.28	3	103.16 ± 5.58	30	100.26 ± 2.58
QCH	13,000	96.33 ± 3.47	45	100.31 ± 1.74	90	97.85 ± 0.77

*Mean of three determinations.

**Mean of three freeze-thaw cycles.

#### Robustness and system suitability parameters

Table 7 summarizes robustness results the low %RSD reflects method robustness after applying the mentioned changes in flow rate, buffer pH and mobile phase composition. A paired t-test was conducted to compare recoveries for each analyte before and after conditions change. The calculated *t* values (shown in Table 7) are less than the tabulated one (for *n* = 6, *t* tabulated = 2.57, considering 95% confidence interval) which indicates that no significant difference was observed before and after changes made to asses robustness. Hence, the proposed method is robust based on changes made in flow rate, buffer pH and organic strength. The effect of the applied changes on chromatographic performance was evaluated. Increasing the flow rate to 1.1 mL/min decreased the retention times of the three analytes by approximately 0.2 min. While, decreasing methanol concentration in mobile phase by 2% decreased resolution to be 4.66 and 7.12 for DAC and LCV peaks, respectively. Other changes didn’t show observed variations in method performance.

Moreover, system suitability parameters as capacity factor, number of theoretical plates, resolution were calculated. Then compared to guideline values and found to be satisfactory showing good performance of the optimized system as demonstrated in Table 8.

**Table 7:** Robustness results of HPLC determination of each analyte QCM (700, 25 and 50 ng/ml for FTC, DAC and LDV, respectively) in human urine.

Parameter		Emtricitabine (FTC)	Daclatasvir (DAC)	Ledipasvir (LDV)
Flow rate (± 0.1 mL/min)	%RSD	0.65	0.36	0.79
	t value	0.61	0.54	0.67
Buffer pH (± 0.2)	%RSD	1.40	0.65	0.47
	t value	0.87	0.63	0.55
Organic strength (± 2%) in methanol	%RSD	1.39	1.01	0.57
	t value	0.84	0.76	0.59

**Table 8:** System suitability parameters of HPLC determination of emtricitabine (FTC), Daclatasvir (DAC) and Ledipasvir (LDV) in human urine.

Parameters	Emtricitabine (FTC)	Daclatasvir (DAC)	Ledipasvir (LDV)	Reference values^41^
Retention time (min)	2.60	4.80	9.89	-
Tailing factor (T)	1.33	1.36	1.15	≤ 2
Number of theoretical plates (N)	2578	3366	3326	> 2000
Capacity factor (k’)	1.17	3	7.25	1–10
Resolution (R_s_)	−	4.73	7.32	> 2
Selectivity (α)	−	2.56	2.42	1–5

## Conclusion

The three co-administered drugs; FTC, DAC and LDV used for HIV/HCV co-infection were quantified in human urine by our validated method. It was validated according to the FDA bioanalytical validation guidelines. The method achieved good accuracy, precision and sensitivity over wide concentration range. It is the first reported method for simultaneous determination of the studied drugs. We propose applying the method for routine urine analysis of HIV/HCV co-infected patients aiming to monitoring the tested drugs levels and ensuring patients’ drug adherence. The significance of HIV patients’ obedience to their drug regimen highly impacts the global public health and lessens threats to the communities.

## Data Availability

Data is provided within the manuscript and any other required details can be furnished by the corresponding author upon request.
